# BioMonitor 2 in-office setting insertion safety and feasibility evaluation with device functionality assessment: results from the prospective cohort BioInsight study

**DOI:** 10.1186/s12872-020-01439-8

**Published:** 2020-04-15

**Authors:** Khaled Awad, Raul Weiss, Asim Yunus, Jon M. Bittrick, Rajasekhar Nekkanti, Mahmoud Houmsse, Toshimasa Okabe, Teagan Adamson, Crystal Miller, Abdul K. Alawwa

**Affiliations:** 1 Mercy Clinic Heart and Vascular at Mercy Heart Hospital, St. Louis, MO USA; 2 The Ohio State University Wexner Medical Center, Columbus, OH USA; 3 Michigan CardioVascular Institute, Saginaw, MI USA; 4 Upstate Cardiology, Greenville, SC USA; 5 The Brody School of Medicine at East Carolina University, Greenville, North Carolina USA; 6 BIOTRONIK, Inc, Lake Oswego, Oregon USA; 7 Cardiology Consultants of East Michigan, Flint, MI USA

**Keywords:** Insertable cardiac monitor, Office procedure, Safety, Feasibility, BioMonitor 2, Adverse event

## Abstract

**Background:**

Insertable cardiac monitors are utilized for the diagnosis of arrhythmias and traditionally have been inserted within hospitals. Recent code updates allow for reimbursement of office-based insertions; however, there is limited information regarding the resources and processes required to support in-office insertions. We sought to determine the safety and feasibility of in-office insertion of the BioMonitor 2 and better understand in-office procedures, including patient selection, pre-insertion protocols, resource availability, and staff support.

**Methods:**

Patients meeting an indication for a rhythm monitor were prospectively enrolled into this single-arm, non-randomized trial. All patients underwent insertion in an office setting. Two follow-up visits at days 7 and 90 were required. Information on adverse events, device performance, office site preparations, and resource utilization were collected.

**Results:**

Eighty-two patients were enrolled at six sites. Insertion was successful in all 77 patients with an attempt. Oral anticoagulation was stopped in 20.8% of patients and continued through insertion in 23.4%, while prophylactic antibiotics were infrequently utilized (37.7% of study participants). On average, the procedure required a surgeon plus two support staff and 35 min in an office room to complete the 8.4 min insertion procedure. The mean R-wave amplitude was 0.77 mV at insertion and 0.67 mV at 90-days with low noise burden (2.7%). There were no procedure related complications. Two adverse events were reported (event rate 2.7% [95% CI 0.3, 9.5%]).

**Conclusions:**

In-office insertion of the BioMonitor 2 is safe and feasible. Devices performed well with high R-wave amplitudes and low noise burden. These results further support shifting cardiac monitor insertions to office-based locations.

**Trial registration:**

clinicaltrials.gov, NCT02756338. Registered 29 April 2016.

## Background

Insertable cardiac monitors (ICMs), also referred to as implantable loop recorders, are utilized when long term monitoring is needed for patients with syncope, cryptogenic stroke, infrequent arrhythmias, and for follow up after cardiac ablation procedures [1]. Traditionally, ICMs have been inserted in a laboratory or operating room setting owing to the relatively larger size of their initial iterations and availability of reimbursement codes for hospital-based insertions. Newer miniaturized device designs and specialized insertion tools simplified the associated procedure making the office setting appealing for insertion [2].

Newly approved procedure codes for office-based insertion reimbursement through Centers for Medicare and Medicaid Services is anticipated to shift some ICM insertions to physician office spaces. Based on prior reports, patients are usually more satisfied with office based surgery because of the convenience and lack of delays [3, 4]. A study compared the safety of ICM insertion in an office setting to those performed in a hospital [5]. That study showed no significant difference in safety or efficacy. Additionally, physicians and patients felt the office setting was more convenient.

The BioMonitor 2 (BioMonitor 2-AF, BIOTRONIK SE & Co. KG, Berlin, Germany) is a programmable ICM that provides information on the occurrence of arrhythmias and records subcutaneous ECGs via two electrodes on opposite ends of the device. The device is approximately 88 mm in length, 4.3 cc in volume, and consists of a rigid body with a flexible tip. The device is placed in the subcutaneous space through a small incision on the left side of the chest wall after infiltration with a local anesthetic. The BioMonitor 2 has previously been shown to provide excellent sensing with consistent sensing over time and low noise [6, 7].

In this single-arm, nonrandomized study, we sought to determine the safety and feasibility of in-office insertion of the BioMonitor 2. Additionally, we aimed to assess site preparation requirements and resource utilization when shifting ICM insertions to the office setting.

## Methods

### Study design

The BioInsight study (ClinicalTrials.gov identifier: NCT02756338) was a multi-center, prospective, unblinded, post-market single-arm study. The study was approved by the institutional review board at each participating site (Western IRB, The University of Kansas Medical Center Human Research Protection Program IRB, and East Carolina University and Medical Center IRB). The study was conducted in accordance with U.S. Federal regulations and local legal and regulatory requirements. Potential patients were identified by the physician investigators from their general patient population at the approved study site. Patients indicated for continuous arrhythmia monitoring with an ICM who were willing to undergo the insertion procedure in an office setting were invited to participate and written informed consent was obtained prior to study procedures. Patients with a compromised immune system or at high risk of developing infection, as identified by the study physician, infection within the last 30 days, or international normalized ratio (INR) greater than 3.5 (assessed within 7 days of insertion for all patients on warfarin) were excluded. Patients were considered fully enrolled once an in-office insertion with a BioMonitor 2 at an approved study site was attempted by a study investigator beginning with the application of local anesthesia. For this study, office setting was defined as a designated area wherein the patient can lay flat and excluded operating rooms, cardiac catheterization laboratories, and electrophysiology laboratories. All devices were donated by the sponsor as reimbursement for office-based insertion was not common at the time of this study.

Fully enrolled patients were required to complete in-clinic visits at approved study sites for a wound check visit at 7 days after the procedure and a follow-up visit at 90 days post-insertion. Measured device data, including R-wave amplitude and noise burden (percentage of time non-physiologic events were sensed by device per day), were collected via remote daily monitoring and from device interrogation at required clinic visits. Information on adverse events, insertion procedure characteristics, and resource utilization was reported by participating study sites. Patients with an unsuccessful or aborted insertion procedure were planned to be followed for 30 days post-insertion to capture any complications related to the insertion procedure and then exited from the study.

Physicians completed at least two procedures in their standard setting prior to study participation. Insertion techniques were based on physician preference and supplies were obtained at the discretion of the study site. Required resources and supply utilization surveys were collected from all investigative sites.

It was anticipated that at least 75 individual patients would be enrolled, undergo a single in-office insertion attempt at a clinic visit that occurred within 30 days of written informed consent, and then be followed through 90 days post-insertion. Due to the descriptive nature of the study, sample size calculations were not performed using power calculations as no hypothesis test was conducted. Instead, the sample size was determined by the proportion of expected adverse events requiring invasive intervention in combination with an exact binomial one-sided upper 95% confidence interval. The primary objective of the study was to characterize all insertion procedure-related complications requiring invasive intervention to resolve. Potential adverse events were identified through a literature search and pre-defined within the protocol. Insertion procedure-related complications included but were not limited to non-healing device pocket, infection with device pocket identified as primary source, and excessive bleeding. Device-related complications included device migration, skin erosion at site of device, and device protrusion. Infections with a secondary source (i.e., not from the device pocket) were collected and defined as non-procedure, non-device related. An independent Clinical Events Committee consisting of 3 electrophysiologists was responsible for reviewing and adjudicating all reported adverse events.

Secondary objectives included characterization of all insertion procedure-related adverse events (including those not requiring invasive intervention), characterization of the insertion procedure (specifically, insertion success, inserted device orientation, final incision size, and total procedure duration), and characterization of device reported R-wave amplitudes throughout the study duration. Additional data of interest included patient demographics, indication for ICM insertion, prescription of pre- and post-procedure antibiotics, oral anticoagulation usage, overall adverse event rate, and resources used during the insertion procedure.

### Statistical analysis

All analysis was intention to treat and included all study participants with a successful insertion and any subject in which the insertion was unsuccessful due to a protocol defined procedure-related adverse event or the insertion procedure was aborted after local anesthesia was applied.

As the study included no pre-defined hypothesis, data analyses were limited to descriptive statistics to present and summarize the data collected in the clinical study. Adverse events were assessed by performing exact, point estimates and one-sided upper 95% confidence intervals. Frequency distributions and cross tabulations are presented for discrete variables. Means, standard deviation, and ranges are presented for continuous variables. SAS version 9.2 (SAS Institute Inc., Cary, NC) was utilized.

## Results

### Study population

**Fig. 1:**
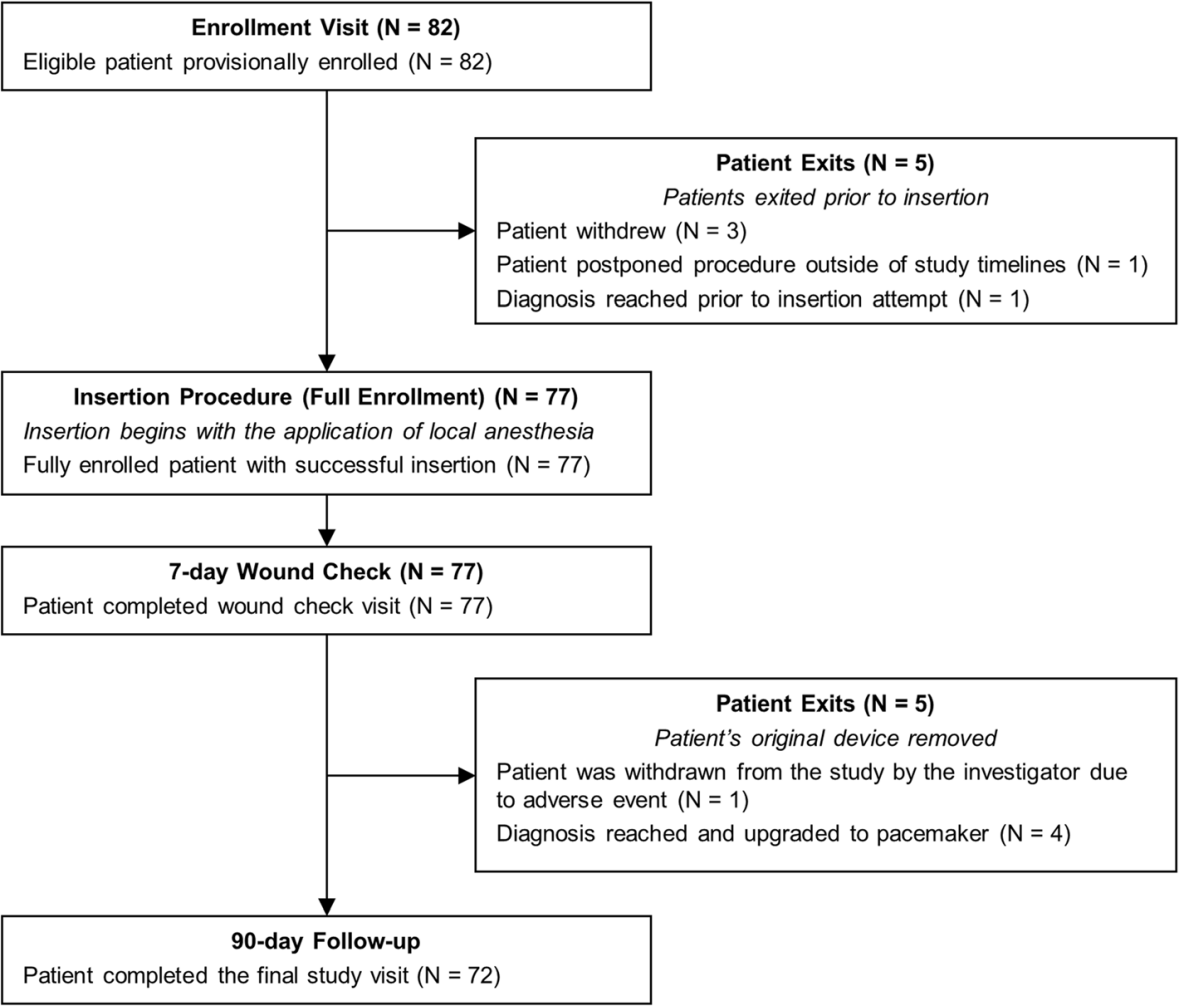
Flowchart of Patient Enrollment and Study Participation

Eighty-two patients were enrolled at six sites between November 7, 2016 and March 13, 2017 with last patient visit completed on July 4, 2017. Nine different physicians attempted device insertion in 77 patients, all of which occurred in an office setting and were successful. Five patients exited from the study prior to insertion attempt and an additional five exited prior to their 90-day follow-up visit. A flowchart of study participation is included in Fig. 1.

Baseline characteristics are summarized in Table 1. The most common primary indication for insertion was management of known atrial tachycardia or atrial fibrillation at 48.1%. A majority of patients had a past history of atrial fibrillation (53.2%) and hypertension (62.3%). There was no evident differences in baseline characteristics for patients who completed the study and those who exited prior to the 90-day follow-up visit.

**Table 1:** Baseline demographic and primary indication for fully enrolled patients

Patient Characteristics	Patients ***N*** = 77
Age at Enrollment (years ± SD)	62.1 ± 15.4
Gender	
Male	41 (53.2%)
Female	36 (46.8%)
Height (in ± SD)	67.8 ± 4.8
Weight (lbs ± SD)	205.6 ± 7.9
BMI (kg/m^2^ ± SD)	31.4 ± 6.6
Race	
White	71 (92.2%)
Black or African American	5 (6.5%)
Not Specified	1 (1.3%)
Primary Indication for Insertion	
Management for known AT/AF	37 (48.1%)
Diagnosis of unexplained syncope	17 (22.1%)
Palpitations	8 (10.4%)
Diagnosis of suspected AT/AF	6 (7.79%)
Cryptogenic Stroke	4 (5.19%)
Near syncope	3 (3.90%)
Other cardiac arrhythmia	2 (2.60%)
Other Medical History	
AF	41 (53.2%)
Atrial Flutter	14 (18.2%)
Bradycardia	19 (24.7%)
Cardiomyopathy	10 (13.0%)
Hypertension	48 (62.3%)
Hypotension	3 (3.9%)
Syncope	20 (26.0%)
Ventricular Tachycardia	5 (6.5%)
Other AT	10 (13.0%)
History of Ablation	31 (40.3%)
Ablation for AF	21 (27.3%)
Ablation for Atrial Flutter	6 (7.79%)
Ablation for Other AT	3 (3.90%)
Ablation for Ventricular Tachycardia	1 (1.3%)

Values are presented as n (%) unless otherwise indicated. *AF* atrial fibrillation, *AT* atrial tachycardia

### Insertion procedure

**Fig. 2:**
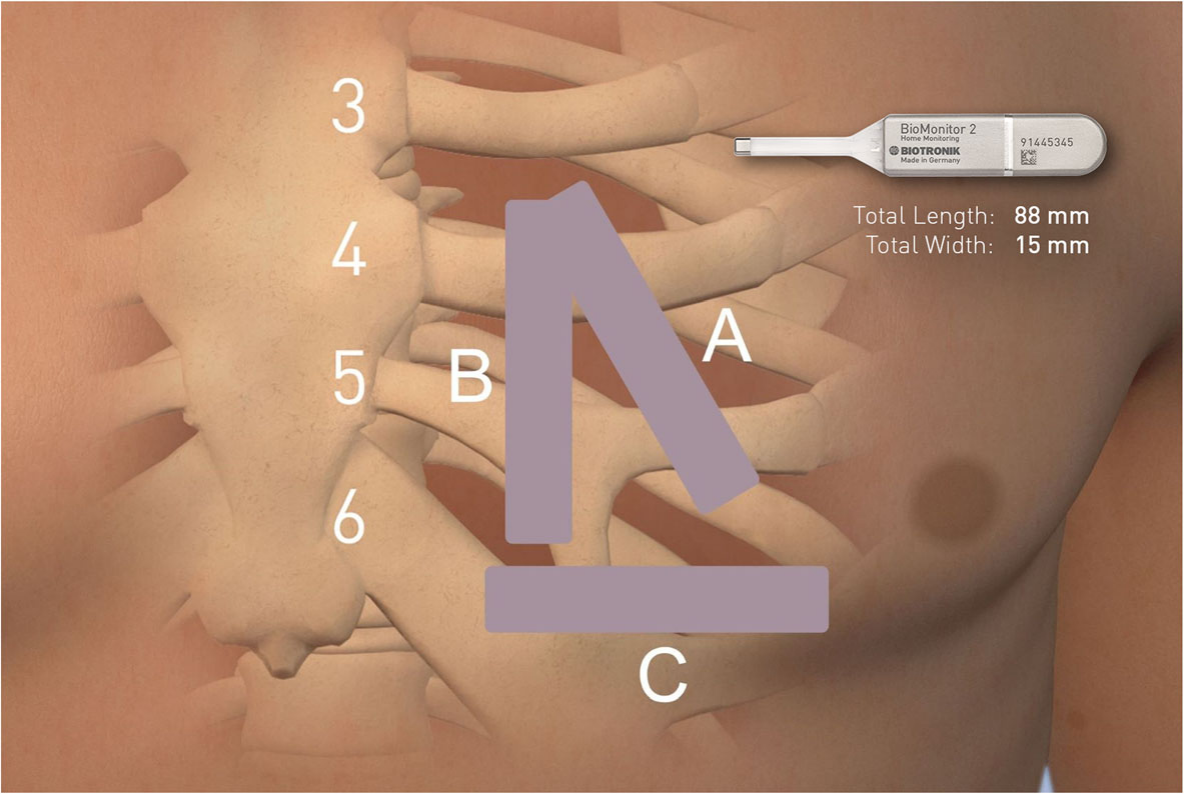
Standard Insertion Positions of the BioMonitor 2. Position A: 45°relative to the sternum over the fourth intercostal space. Position B: parallel to the sternum over the fourth intercostal space. Position C: perpendicular to the sternum, sub-mammary

Characteristics of the insertion procedures are displayed in Table 2. A total of 34 out of 77 patients (44.2%) were taking oral anticoagulants at study enrollment. Prior to the insertion procedure, 16 participants had their oral anticoagulant medication held from one to 4 days with a median of 1 day. A majority of the total study participants (62.3%) did not receive prophylactic antibiotic therapy. No patients received intravenous medications during the insertion procedure and electrocautery was not required for any study insertion. At insertion, the majority of devices were oriented in either position A (45°relative to the sternum over the fourth intercostal space, 45.5% of insertions) or position B (parallel to the sternum over the fourth intercostal space, 49.4% of insertions), as opposed to position C (perpendicular to the sternum, sub-mammary) and other positions. Standard insertion positions are depicted in Fig. 2.

**Table 2:** Insertion procedure characteristics for fully enrolled patients

Characteristic	Patients ***N*** = 77
Device Orientation	
Position A	35 (45.5%)
Position B	38 (49.4%)
Position C	1 (1.3%)
Other	3 (3.9%)
Final Incision Size (mm ± SD)^a^	14.9 ± 3.7
Total Procedure Duration (min ± SD)	8.4 ± 3.7
Prophylactic Antibiotic Use	29 (37.7%)
After the procedure^b^	29 (37.7%)
Before the procedure	3 (3.9%)
Oral Anticoagulant Use	34 (44.2%)
Non-vitamin-K OAC	30 (39.0%)
OACs not held prior to procedure	18 (23.4%)
Closure Material^b^	
Deep tissue (subcutaneous) sutures	50 (64.9%)
Superficial (dermal) sutures	23 (29.9%)
Barbed sutures	13 (16.9%)
Topical adhesive	48 (62.3%)
Skin closure strips	5 (6.5%)

Values are presented in n (%) unless otherwise indicated. OAC, oral anticoagulants. ^a^3 patients missing final incision size. ^b^More than one closure material may have been used

In addition to the surgeon, each procedure was supported by one to three staff members (i.e. nurse, technician, or certified medical assistant). Office rooms were occupied from 20 to 60 min (median 35 min), including preparation and final cleanup. Procedure duration decreased with increased number of insertions from a mean of 10.8 min for first in-office procedure completed by a physician to 6.6 min by the sixth procedure completed by the same physician (overall mean 8.4 min).

Six of the seven study sites reported procedure supply costs which averaged $130 per patient (range $36 to $219). The standard supplies utilized are listed in Table 3. Additionally, two sites had a hand-held cautery device available; however, cautery was not used during the study.

**Table 3:** Typical procedure supplies for in-office insertion

Category	Supplies
Surgical attire	Cap, gown, gloves, mask
Room requirements	Exam table, tray, table/tray cover
Skin preparation	Patient drape, chlorhexidine topical cleanser with applicator or scrub brush, marker
Anesthetic	Syringe with needle, anesthetic (specifically, lidocaine-epinephrine or lidocaine alone)
Incision	Scalpel, sponge gauze
Insertion	Insertion tool set (included with ICM)
Wound closure	Forceps, suture needle with or without driver and counter, sutures, scissors, topical skin adhesive/strips or wound dressing

### Device functionality

**Fig. 3:**
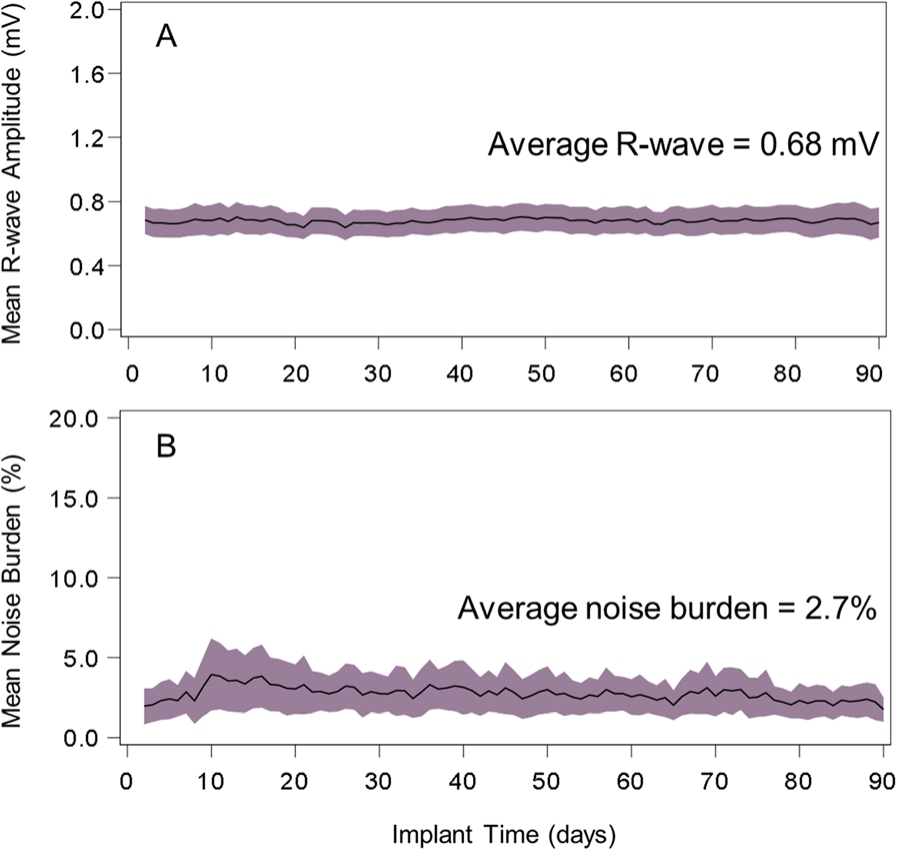
Long Term Amplitude and Noise Trends. The mean and 95% CI for long term (**a**) R-wave amplitude and (**b**) noise burden trends extrapolated from all study partcipants using BIOTRONIK Home Monitoring® data are presented

The mean R-wave amplitude was 0.77 ± 0.5 mV at insertion (*N* = 77) and 0.67 ± 0.3 mV at the 90-day visit (*N* = 72). The noise burden was 2.7% ± 5.8% at the 90-day visit (*N* = 72). Long term trends for the R-wave amplitude and noise burden were extracted via daily BIOTRONIK Home Monitoring®. Values were similar to discrete data collected at study visits (Fig. 3). Once study participants began transmitting (*N* = 76), the daily transmission rate for the study was 93.7%.

### Adverse events

Among the 77 patients enrolled, there were no complications related to the insertion procedure (0.0%; 95% CI 0.0, 5.0%). In total, two adverse events were reported and adjudicated by the Clinical Events Committee resulting in an overall event rate of 2.7% (95% CI 0.3, 9.5%). One adverse event resulted from an allergic reaction to surgical adhesive which resolved spontaneously. The other adverse event involved device protrusion and a delayed secondary infection. The device eventually dislodged out of the pocket 77 days after insertion without surgery or invasive intervention; therefore, the Clinical Events Committee determined this event did not meet the definition of a procedure related complication due to the secondary source of infection.

## Discussion

There is limited data to support the safety of ICM insertion in the office setting [5]. In this study, all insertion attempts were successful and the procedures were completed quickly. There were no acute complications related to the procedure and the overall event rate was 2.7%. The insertion success rate of 100% and low adverse event rate reported for the BioMonitor 2 is consistent with prior reported values of 2.9% in an office and 4.4% in the hospital for other ICM devices [5].

Patient selection and site preparation are important factors when considering office based insertions of ICMs [8]. ICM insertions inherently have a low bleeding risk due to the small device size and subcutaneous placement [9]. Therefore, only patients at increased bleeding risk, specifically those on warfarin with a recent high international normalized ratio (INR), were not considered candidates for the study. No bleeding events, such as hemorrhage or hematoma, were reported despite 18 patients (23.4%) continuing oral anticoagulant therapy at the time of insertion.

Infection risk was mitigated by excluding patients with recent infection and those with compromised immune systems, as well as use of sterile techniques during insertions. In this study, the infection rate was low and similar to previously reported rates for hospital based insertions (0 to 1.6% in studies of 122 to 375 participants) [5, 10, 11]. Investigators chose to utilize prophylactic antibiotics, primarily post-procedure, for 38% of the study patients. Pre-procedure antibiotics have frequently been used in similar studies (31–100%) [5, 11, 12], though guidance for prophylactic antibiotic usage during ICM insertion has not been established [13].

For minimally invasive cardiac procedures, an office or clinic room has been utilized as an alternative to hospital cardiology procedure spaces [5, 8, 14]. The advantages of moving low risk procedures to an in-office setting include reduced wait times, lower facility fees, and decreased burden on highly trained surgical suite personnel [8, 15, 16]. For select patients with a low risk profile, in-office based insertions should be considered.

### Study limitations

Our study was non-randomized which does not allow for comparison to a control group of insertions performed in a laboratory or operating room setting and is susceptible to bias. Arguably, that was not very critical given extremely low adverse event rate. The follow-up period of 90 days post-insertion may miss late-onset complications, such as delayed infection. The decision to hold OAC and the use of prophylactic antibiotics was not defined in the protocol and varied between physicians. Finally, this observational study was not powered to determine statistical significance in the findings.

## Conclusion

While non-randomized, the BioInsight study supports in-office insertions of the BioMonitor 2 under local anesthesia as a safe and feasible for selected patients. A low adverse event rate was observed, all attempted insertions were successful, and there were no significant procedure related infections despite infrequent use of preoperative antibiotics. Our study further supports shifting ICM insertions to office-based locations and provides information to define site-specific patient selection, pre-insertion processes, and resources needed for these procedures.

## Data Availability

Individual participant data cannot be publicly shared as the patient consent form only allows publication of aggregated data. Requests to access de-identified participant level data should be directed to BIOTRONIK Clinical Studies (BIOTRONIK, Inc., Attn: Clinical Studies, 6024 Jean Road, Lake Oswego, OR; 1–800–547-0394). To gain access, the requesters will need to sign a data use/access agreement and agree that only aggregated data will be published. Requesters will be granted access to the same data sets that were used for the analysis presented in this publication.

## Abbreviations

ICM: Insertable cardiac monitor
INR: International normalized ratio
